# Decision Tree for Key Comparisons

**DOI:** 10.6028/jres.126.007

**Published:** 2021-04-27

**Authors:** Amanda Possolo, Amanda Koepke, David Newton, Michael R. Winchester

**Affiliations:** 1 National Institute of Standards and Technology, Gaithersburg, MD 20899, USA; 2 National Institute of Standards and Technology, Boulder, CO 80305, USA

**Keywords:** Bayesian, Cochran's *Q* test, degrees of equivalence, Gaussian, hierarchical, key comparison reference value, Laplace, Shapiro-Wilk test, skew-*t*, Student, symmetry test, weighted average, weighted median

## Abstract

This contribution describes a Decision Tree intended to guide the selection of statistical models and data reduction procedures in key comparisons (KCs). The Decision Tree addresses a specific need of the Inorganic Analysis Working Group (IAWG) of the Consultative Committee (CC) for Amount of Substance, Metrology in Chemistry and Biology (CCQM), of the International Committee for Weights and Measures (CIPM), and it is likely to address similar needs of other working groups and consultative committees.

Because the portfolio of KCs previously organized by the CCQM-IAWG affords a full range of opportunities to demonstrate the capabilities of the Decision Tree, the majority of the illustrative examples of application of the Decision Tree are from this working group. However, the Decision Tree is widely applicable in other areas of metrology, as illustrated in examples of application to measurements of radionuclides and of the efficiency of a thermistor power sensor.

The Decision Tree is intended for use after choices will have been made about the measurement results that qualify for inclusion in the calculation of the key comparison reference value (KCRV), and about the measurement results for which degrees of equivalence should be produced. Both these choices should be based on substantive considerations, not on purely statistical criteria. However, the Decision Tree does not require that the measurement results selected for either purpose be mutually consistent.

The Decision Tree should be used as a guide, not as the sole and autonomous determinant of the model that should be selected for the measurement results obtained in a KC, or of the procedure that should be employed to reduce these results. The scientists running the KCs ultimately have the freedom and responsibility to make the corresponding choices that they deem most appropriate and that best fit the purpose of each KC.

The Decision Tree involves three statistical tests, and comprises five terminal leaves, which correspond to as many alternative ways in which the KCRV, its associated uncertainty, and the degrees of equivalence (DoEs) may be computed.

This contribution does not purport to suggest that any of the KCRVs, associated uncertainties, or DoEs, presented in previously approved final reports issued by working groups of the CCs should be modified. Neither do the alternative results question existing, demonstrated calibration and measurement capabilities (CMCs), nor do they support any new CMCs.

## 1 Introduction

Key comparisons (KCs) are a particular kind of interlaboratory study intended to characterize quantitatively the degree of equivalence of national measurement standards. The participants in KCs are national metrology institutes (NMIs), or their designates, of countries that are signatories of the arrangement for the mutual recognition (MRA) of national measurement standards and of calibration and measurement certificates issued by the NMIs. The MRA was drafted by the International Committee for Weights and Measures (CIPM) under the authority given to it in the Metre Convention [1].

In the context of this arrangement, the degree of equivalence (DoE) of measurement standards is taken to mean the degree to which these standards are consistent with reference values determined from the KCs and hence are consistent with one another. The DoE of a national measurement standard is expressed quantitatively in terms of its deviation from the key comparison reference value (KCRV) and the uncertainty of this deviation.

The MRA does not specify how KCRVs should be computed or how their uncertainties should be evaluated. The MRA (in the 2003 revision of its Technical Supplement) explains that "the degree of equivalence of each national measurement standard is expressed quantitatively by two terms: its deviation from the key comparison reference value and the uncertainty of this deviation (at a 95% level of confidence). The degree of equivalence between pairs of national measurement standards is expressed by the difference of their deviations from the reference value and the uncertainty of this difference (at a 95% level of confidence)" [1, Sec. T.2].

The most common and simplest form of a KC organized by the Inorganic Analysis Working Group (IAWG) of the Consultative Committee for Amount of Substance, Metrology in Chemistry and Biology (CCQM), involves measurement and intercomparison of measurement results of the same measurand in different aliquots of the same material distributed to the participants, where the material has been selected to demonstrate specific measurement capabilities, and its homogeneity and stability have been characterized in advance of the KC.

The measurement capabilities to be demonstrated may inherently include methodεs of sample preparation and extraction of the element or compound of interest, ability to cope with challenges posed by the matrix containing the analyte, application of methods of analysis, delineation of credible, realistic uncertainty budgets, establishment of meaningful metrological traceability, and execution of the calculations necessary to produce an estimate of the measurand, and to characterize the associated uncertainty.

The uncertainty associated with each measured value *y*, as evaluated and reported by each participant in the KC, may be expressed as an expanded uncertainty *U_p_*(*y*), with specified coverage probability 0 *< p <* 1, with the understanding that the interval *y±U_p_*(*y*) is believed to include the true value of the measurand with probability *p*. Alternatively, the uncertainty may be expressed as a (combined) standard uncertainty, *u*_c_(*y*), with the understanding that it represents the standard deviation of a probability distribution that describes the uncertainty surrounding the estimate of the measurand.

The uncertainty (expanded or standard) may be expressed using an asymmetrical interval, a fully specified probability distribution, or a sample drawn from the probability distribution that describes the uncertainty surrounding the KCRV or difference in the DoE. These alternatives have not seen much use in KCs, but have otherwise been used in measurement science [2-5] and in specific scientific disciplines [6].

Ideally, and according to Ref. [7, p. 14], each reported uncertainty (however it may be expressed), should be qualified with the number of degrees of freedom that support it, in accordance with Annex G of the *Guide to the Expression of Uncertainty in Measurement* (GUM) [8]. This requirement notwithstanding, numbers of degrees of freedom often are not reported. However, when they are reported, the procedures specified in the leaves of the Decision Tree described in Sec. 2 will take them into account.

A cursory examination of final reports of KCs organized by the CCQM-IAWG reveals that KCRVs and DoEs have historically been computed in several different ways, and considerable time and effort have regularly been expended discussing and finally selecting particular ways in which the KCRV and the DoEs have been computed.

The CCQM-IAWG is not alone in this endeavor, for other CCs of the CIPM have faced the same challenges, and so have much larger communities concerned with similar comparisons, conducted for a wide range of purposes.

The largest such community, which is also the one that publishes the largest number of results from comparative studies per year, is the medical community [9] where, in contrast with KCs, different measurements are not planned in advance and performed in coordination, but only their results are blended *a posteriori*, typically after they will have been published independently of one another. For this reason, they are often described as "meta-analyses' [10].

The medical and other communities that seek to bolster confidence in research results by blending information from multiple studies face challenges similar to those that the CCs face [11-13], and similar quandaries [14]. Meta-analysis has made very important contributions to human health and public health policy [15-17].

Section 2 outlines the approach to the development of the Decision Tree, including a review of principles originally introduced in Ref. [18] that inform the models and procedures for data reduction implemented in the Decision Tree. Section 3 describes these models and procedures: adaptive weighted average, weighted median, and three Bayesian, hierarchical models responsive to different features of the measurement results. Section 4 provides examples of use, in the form of reanalyses of historical measurement results from selected comparisons organized by different CCs. Section 5 summarizes the lessons learned from the examples, and offers recommendations for the use of the Decision Tree in future KCs.

## 2 Decision Tree and Guiding Principles

**Fig. 1:**
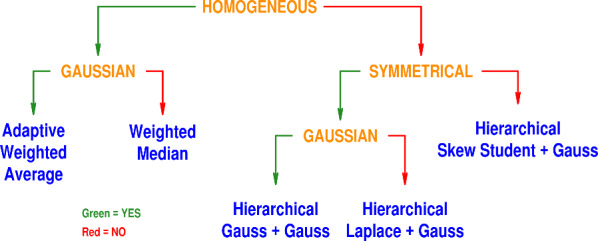
The Decision Tree comprises four branching nodes (orange) and five leaves (blue) that suggest different models for the measurement results and corresponding procedures for data reduction. A question needs to be answered at each node: if the answer is YES, then one follows the green branch (toward the left); if the answer is NO, then one follows the red branch (toward the right), until one reaches a leaf.

Figure 1 depicts the Decision Tree, which comprises four branching nodes (orange) and five leaves (blue). The leaves indicate different procedures for data reduction, each of which has an underlying statistical model. To use the Decision Tree one answers a question at each node, and follows the course corresponding to the answer (YES or NO), until one reaches a leaf.

The Decision Tree reflects the current state of the statistical arts in modeling and data analysis for KCs and other interlaboratory studies and meta-analyses. It represents a compromise among rigor, simplicity, and practicability, offering a reasonable, general-purpose solution to the recurring problem of how to reduce the measurement results obtained in a KC, best to support the mutual recognition of national measurement standards and of calibration and measurement certificates issued by national metrology institutes (NMIs) [1].

The solutions offered by the Decision Tree express a subjective understanding of the principal issues facing the CCs as they set out to model and reduce data from KCs. These solutions are not unique because in most cases there will be alternative, similarly rigorous solutions that could be adopted. However, in addition to its simplicity and practicability, the Decision Tree is also consistent with the general principles for model selection and data reduction in interlaboratory studies enunciated by Koepke *et al.* [18], which we summarize and reformulate as follows, to make their intent clear in the present context:

(**P1**) It is the prerogative and responsibility of the organizing CC or working group to select the measurement results that will determine the KCRV. However, DoEs will be computed for all participants regardless of whether or not their results are included in the calculation of the KCRV and associated uncertainty, unless they will have chosen to withdraw from the KC. The selection of some results for the computation of the KCRV, and the exclusion of others, should be based on substantive reasons that are documented in the KC final report. The mere fact that a measured value lies far from the bulk of the others alone is insufficient reason to set it aside, even if a statistical test suggests that it is an "outlier".

(**P2**) No measured value should dominate the consensus value "automatically," simply because the associated measurement uncertainty is much smaller than the uncertainties associated with the other measured values.

To this end, procedures for data reduction should include some damping mechanism to limit the influence that measured values with unusually small associated uncertainties will have upon the KCRV. This provision is consequential only when the measurement results are markedly mutually inconsistent: that is, when the measured values are significantly more dispersed than the reported uncertainties suggest that they should be.

(**P3**) Measurement methods should be sufficiently well characterized to warrant confidence in the belief that the measured values, taken as a group, are roughly centered at the true value of the measurand. Participating laboratories should have previously demonstrated sufficient competence based on satisfactory performance in previous KCs, pilot studies, or proficiency tests.

If all the measured values would tend to be too low or too high relative to the true value of the measurand, no statistical procedure that relies on the data alone will be able to detect this and "correct" the consensus estimate accordingly, but the DoEs may, even in such cases, still be comparable and informative.

(**P4**) A model should be formulated that explicitly relates the measured values to the true value, *µ*, of the measurand, and that includes elements representing contributions from all recognized sources of uncertainty. Furthermore, the estimation of *µ*, and the evaluation of the associated uncertainty, should be consistent with the statistical model and with some principle of estimation whose general reliability is widely recognized.

The calculation of the KCRV is contingent on such a model and on the choice of optimality criterion that the KCRV is intended to satisfy. Suppose that the model specifies that the measured values are equal to the measurand plus random, mutually independent, Gaussian measurement errors. This alone does not suffice to justify combining the measured values in a particular way (say, as weighted average of the measured values). An additional criterion is needed: for example, one that seeks to minimize the mean squared error of the KCRV, or one that requires a KCRV with minimal absolute error. Therefore, the choices that one needs to make to determine the KCRV should take into account the purpose that the KC is intended to serve.

The estimates of the parameters in the model, and derivative quantities -- including the KCRV, associated uncertainty, and DoEs -- ought to be smooth, slowly varying functions of the measurement results. This last requirement applies to individual models underlying the leaves of the Decision Tree, not necessarily to the Decision Tree as a whole. In particular, it speaks against procedures that involve outright rejection of some measurement results instead of a modulated, smooth down-weighting scheme.

(**P5**) The statistical model underlying data reductions should be able to detect, evaluate, and propagate *dark uncertainty* [19], which manifests itself as dispersion of the measured values in excess of what the reported uncertainties suggest that it should be, and thereby accounts for mutual inconsistency of the measurement results.

Failure to recognize and propagate dark uncertainty generally yields DoEs with uncertainties that are too small. Both the average and the median of the measured values, with or without the conventional weights (which are propoεrtional to the reciprocal, squared reported uncertainties) ignore dark uncertainty, and thus tacitly assume that the results are mutually consistent.

The GUM [8] stipulates that the uncertainty associated with a measured value should reflect the contributions of all sources of uncertainty. This implies not only that the reported uncertainties should play a role in the calculation of the KCRV and of the DoEs, but also that the contribution from dark uncertainty should be recognized and propagated.

Even though the uncertainties reported by KC participants may be imperfect, using them is still better than ignoring them. Most software implementations of procedures to reduce data from interlaboratory studies and meta-analyses require that the reported uncertainties be specified or that sufficient data be provided from which the uncertainties of the measured values may be derived: for example, RevMan (https://revman.cochrane.org/ from Cochranne Reviews), and the metafor [20] and other, similar packages for the R environment for statistical computing and graphics [21].

(**P6**) Degrees of equivalence (differences between measured values and the consensus value, or between pairs of measured values, qualified with evaluations of associated uncertainty) should be computed consistently with their primary goal of identifying participants with "unusual" results, in the sense that their measured values lie "beyond the range allowed by the model", as suggested by Jones and Spiegelhalter [22] and elaborated by Koepke *et al.* [18, Sec. 6].

The *NIST Consensus Builder* [23], published by the National Institute of Standards and Technology (NIST), heeds this principle, which implies, in particular, that dark uncertainty is recognized when evaluating the uncertainties in the DoEs. This practice follows from the understanding that uncertainty evaluations should reflect contributions from all sources of uncertainty that one is aware of: it does not matter whether such awareness derives from a bottom-up analysis of the measurement system employed by each participant in the KC, or results from a top-down evaluation done collectively, when the individual results (measured values and reported uncertainties) are put on the table and compared [24, Sec. 3f, p. 16].

## 3 Models and Methods

The Decision Tree involves three statistical tests and five models and procedures for statistical data reduction. The tests are of (1) mutual consistency (that is, homogeneity) of the measurement results, (2) symmetry of the measured values, and (3) Gaussian shape of the (standardized) measured values. The models and corresponding procedures are for weighted averages, weighted medians, and three Bayesian, hierarchical, random effects models.

The measurement results of a KC involving *N* participants may be summarized in *N* triplets, (*x*_1_,*u*_c_(*x*_1_), *v*_1_), . *. .*, (*x_N_,u*_c_(*x_N_*), *v_N_*), each of which comprises a measured value *x_j_*, the associated standard uncertainty *u*_c_(*x _j_*), and the number of degrees of freedom *v_j_* on which the standard uncertainty is based, for *j* = 1, *. . ., N*. However, in many cases the {*v_j_*} are not reported, even though the CIPM requires them [7, p. 14].

In general, only *n*⩽*N* of the measurement results are used to produce the KCRV and to evaluate its associated uncertainty, but DoEs are computed for all *N* participants, except for any that may withdraw from the comparison. The number, *n*, of participants whose results contribute to the calculation of the KCRV, is influential for two reasons: first, because KCs are conducted not only for the benefit of the participants and to honor the obligations of the MRA, but also to learn lessons that should be relevant to a whole community of interested parties; and second, because the value of *n* impacts the reliability of the recommendation of the Decision Tree for how the measurement results should be reduced. For both these reasons, the larger the *n*, the more widely applicable and the more reliable the conclusions.

Bender *et al.* [25, p. 389], who have considered the challenges facing the task of blending results from multiple, independent studies, for purposes of evidence synthesis, concluded that "no satisfactory universal method is currently available to perform meta-analyses in the case of very few studies." Even though their focus was primarily evidence synthesis in the medical field, the challenges are very much the same in interlaboratory studies, and KCs in particular, carried out in measurement science. By "very few" they seem to mean *n* smaller than 10.

The limitations and requirements concerning *n* that are imposed by the three statistical tests governing traversals of the Decision Tree will be discussed in Sec. 3.1.1, Sec. 3.1.2, and Sec. 3.1.3. In general, the smaller the *n*, the lower the power of these tests: that is, the smaller the probability of detecting heterogeneity, or asymmetry, or non-Gaussian shape, when in fact they prevail.

According to principle (P1) from Sec. 2, the selection of the measurement results for inclusion in the characterization of the KCRV should be based on substantive considerations, not on statistical criteria. In particular, (P1) rules out the concept of *largest consistent subset* proposed by Cox [26] as a basis for the aforementioned selection.

The generic model for weighted averages and weighted medians is the so-called *common mean* model, which expresses each measured value as *x_j_* = *µ* + *ε_j_*, where *µ* denotes the true value of the measurand, and *ɛ_j_* denotes measurement error.

The hierarchical models all are *random effects* models, which express each measured value as

*x_j_* = *µ* + *ℷ_j_* + *ε_j_,* (1)

where *µ* denotes the true value of the measurand, *ℷ_j_* denotes a participant's effect (which may be positive or negative, according to whether the participant tends to produce high or low values), and *ε_j_* denotes measurement error specific to participant *j*, for *j* = 1, *. . ., n*.

It is possible to distinguish the {*ℷ_j_*} from the {*ε_j_*} that appear in Eq. (1) because the reported uncertainties {*u_c_*(*x_j_*)*}* are also part of the data, not only the measured values. If the {*x_j_*} are more dispersed than the {*u_c_*(*x_j_*)} suggest that they should be, then the {*ℷ_j_*} cannot all be zero.

The random effects {*ℷ_j_*} are modeled as a sample from a probability distribution with mean 0 and standard deviation *τ* that quantifies dark uncertainty. The specific probability distribution chosen for the random effects depends on the model, as explained in Sec. 3.1.

In all the models entertained by the Decision Tree, except for the model that leads to the weighted median as estimate of the KCRV, the measurement errors {*ε_j_*} are modeled as non-observable outcomes of independent, Gaussian random variables, all assumed to have mean 0. Their standard deviations {*σ_j_*} generally may differ from one another. Some of the {*σ_j_*} may be treated as being unknown, with the reported uncertainties {*u_c_*(*x_j_*)} serving as estimates of the corresponding {*σ_j_*}, while others may be treated as being known with full certainty, and hence *σ_j_* = *u_c_(x_j_*). The numbers of degrees of freedom, {*v_j_*}, when they are reported, indicate which are which: a reported uncertainty whose *v_j_* is finite is taken as an estimate of the corresponding *σ_j_*; one whose *v_j_* is practically infinite is regarded as being the actual value of *σ_j_*(however unrealistic this assumption may be).

## 3.1 Statistical Tests

Statistical tests may suggest incorrect decisions, and typically they provide insurance against such eventuality by admitting the possibility of error and by controlling the probabilities of error [27]. For example, the test associated with the root of the Decision Tree may erroneously conclude that the results are heterogenous when in fact they are mutually consistent, or it may fail to diagnose the presence of dark uncertainty. The probability of the former (Type I error) is called the *size* of the test. The complementary probability of the latter (that is, the probability of detecting dark uncertainty when it is present) is called the *power* of the test. Similarly for the other tests.

Consistently with the conventional approach to statistical tests of hypotheses [28], the three tests that determine how the Decision Tree is traversed are constructed to "protect" the hypotheses of mutual consistency, symmetry, and Gaussian shape against erroneously concluding them to be otherwise: this is accomplished by specifying suitably small probabilities for their Type I errors. Typical choices for the sizes of these tests are 1% or 5%, but it behooves the scientists responsible for the comparison to choose a size reflecting a level of risk (in reaching the wrong conclusion) they are prepared to entertain. For Cochran's *Q* test, we recommend a generous allowance for the probability of Type I error, say 10%, because it is more consequential to fail to detect dark uncertainty when it exists, than to conclude erroneously that *τ* = 0.

Traversing the Decision Tree involves carrying out two or three statistical tests. Therefore, if the level of overall risk deemed acceptable for the selection of a leaf is 0 *< α <* 1, then the sizes of the individual tests should be adjusted for the actual multiplicity of testing, because the more often one tests the greater the chances of at least one individual test reaching a wrong conclusion.

On the one hand, the most conservative adjustment, which is also valid under the most general conditions, is to "size" the individual tests so that the probabilities of Type I error add up to *α* -- the so-called *Bonferroni Correction* [29]. On the other hand, the tests are applied sequentially in this case, which may compromise the adequacy of this correction.

The recommended approach, in any case, is to focus on the *p*-values of the tests, and decide whether they are commensurate with acceptable risks of erroneous conclusions, or not. The *p*-value is the probability of observing a value of the test criterion at least as deviant from the expected value as was observed, when the hypothesis under test is true, and such deviation occurs by chance alone, owing to the vagaries of sampling.

When discussing the individual tests below, we will point out that statistical significance need not be the sole determinant of a conclusion (YES or NO) and the corresponding path to take at each node of the Decision Tree. Criteria of substantive significance may legitimately weigh upon the decision as well. None of these tests affords 100% probability of detecting significant heterogeneity, or asymmetry, or non-Gaussian shape for the relevant probability distributions when these conditions prevail. In fact, their power generally will be quite low when the number of participants in the KC is small.

The power of a statistical test of a specified hypothesis is the probability of rejecting this hypothesis when it is false. Since the size and power of a statistical test typically move in opposite directions, the desire to achieve greater power usually comes at the price of increasing the probability of incorrectly rejecting the hypothesis that the test "protects" by design.

For example, suppose that there is either precedent or substantive reason to suspect that the measured values may be arranged asymmetrically around the true value of the measurand. In this case one may choose to reject the hypothesis of symmetry for a larger Type I error than when there are no grounds for such suspicion, thus accommodating the expected conclusion.

Skewness in the distribution of the participants' effects (in random effects models of the kind introduced in Eq. (1) and discussed in Sec. 3.2.3, Sec. 3.2.4, and Sec. 3.2.5) may be a consequence of incomplete extraction of the measurand from its matrix, for example of a heavy metal that is complexed in organic tissue, say arsenic in arsenobetaine [30, 31].

### 3.1.1 Mutual Consistency

**Fig. 2:**
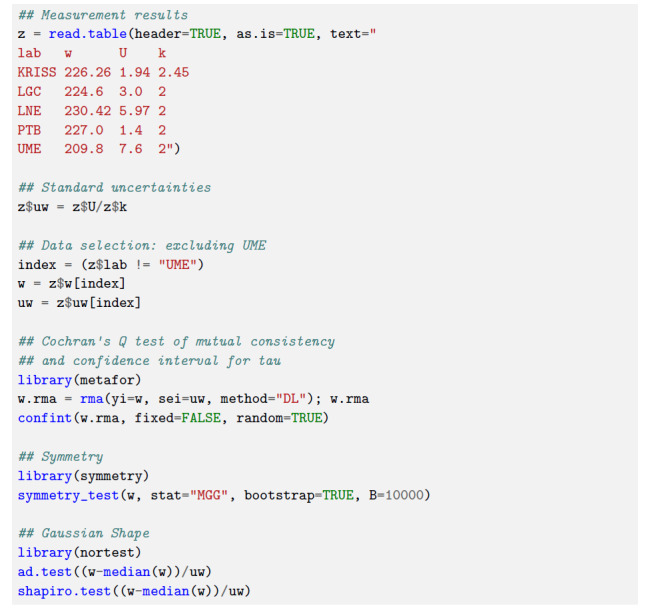
R code implementing Cochrann's *Q* test, the symmetry test described by Miao *et al.* [33], and both the Anderson-Darling [34] and Shapiro-Wilk [35] tests of Gaussian shape, as applied to the measurement results depicts the two versions of the DoEs.rom CCQM-K45 (mass fraction of tin in tomato paste) [36]. UME's measurement result was excluded from the calculation of the KCRV because their analytical procedure deviated significantly from the protocol of the KC. On the one hand, the test statistic for Cochran's test was *Q* = 3.92 which, when compared with the chi-squared reference distribution with three degrees of freedom, yielded a *p*-value of 0.27, thus not rejecting the hypothesis of mutual consistency. On the other hand, the confidence interval for *τ* produced by confint ranges from 0.0 mg*/*kg to 8.8 mg*/*kg, and this upper endpoint amounts to 4% of the KCRV and is eight times larger than the median of the reported standard uncertainties. The symmetry test yielded a *p*-value of 0.13, thus not rejecting the hypothesis that the measured values originate from a symmetric distribution. Both tests of Gaussian shape were applied to the roughly standardized measured values. The Anderson-Darling test ended with an error because its implementation cannot handle samples with fewer than eight observations. The Shapiro-Wilk test produced a *p*-value of 0.92, thus not rejecting the hypothesis of Gaussian shape.

Cochran's chi-squared, or *Q* test, is widely used to detect mutually inconsistent measurement results [32]. Figure 2 lists R code illustrating how this and the other tests involved in traversing the Decision Tree may be applied.

Cochran's *Q* test generally has low power to detect heterogeneity (that is, mutual inconsistency) of the measurement results [37-39], especially for small *n*. Any *n <* 10 should be regarded as "small" in this context. However, power depends also on the magnitude of the dark uncertainty, *τ*, that is invoked to "explain" the heterogeneity: power typically increases with increasing *τ*.

An abundance of caution has motivated us to build a provision into the Decision Tree that lessens the impact of wrong choices regarding homogeneity: even when Cochran's *Q* test does not detect significant heterogeneity and the measured values satisfy the Gaussian assumption, the Decision Tree suggests that an adaptive weighted average be used instead of the conventional weighted average. This adaptive weighted average is the DerSimonian-Laird procedure [18, 40], as will be explained below.

It should be noted that the reason to accept or reject the hypothesis of homogeneity need not be only the *p*-value of the test. It is reasonable also to take into account the actual, relative magnitude of *τ*, the contribution from dark uncertainty. If *τ* (which is a standard deviation) is only a small fraction (say, less than 10%) of the reported standard uncertainties, then it may not be substantively consequential, even if it is statistically significant.

An estimate of the relative magnitude of *τ*, as well as an assessment of its statistical significance, will help the user decide whether there appears to be sufficient, substantively meaningful heterogeneity, and to proceed accordingly. Also, a coverage interval for *τ* is generally more useful and informative than a statistical test. R function confint defined in package metafor can and should be used routinely to produce confidence intervals for *τ*, as illustrated in Fig. 2. This function implements the *Q*-profile likelihood method described in Refs. [41] and [42].

In many cases, the left endpoint of a confidence interval for *τ* will be 0, suggesting that there is no significant contribution from dark uncertainty. However, if the right endpoint amounts to a large proportion of both the measured values and of the reported standard uncertainties, then such suggestion, even when it is reinforced by a large *p*-value from Cochran's *Q* test, should not be accepted automatically.

A Bayesian procedure, for example the *Hierarchical Gauss* + *Gauss* procedure mentioned in Sec. 3.2.3, can provide a large sample from the posterior distribution of *τ*, from which an estimate of the corresponding probability density can be built, for example using R function density. If this density appears to have a single mode (value where it reaches a maximum) clearly away from zero, then this is persuasive evidence in favor of there being heterogeneity. However, carrying out such inquiry involves some offline processing of the optional output of the *NIST Consensus Builder*, for example.

Alternatively, especially when the number of participants is small and the measured values seem to conform with the assumption of Gaussian shape, one may still opt for the hierarchical model with Gaussian random effects and Gaussian errors (third leaf from the left in Fig. 1), instead of the adaptive weighted average, because the hierarchical model propagates dark uncertainty reliably, both to the KCRV and to the DoEs.

Even though one should strive not to fail to detect significant heterogeneity, it should also be noted that when many laboratories are involved, Cochran's *Q* test may detect statistically significant heterogeneity that is substantively irrelevant [43]. Hoaglin [39] reviewed these and other shortcomings of the test.

### 3.1.2 Symmetry

The test proposed by Miao *et al.* [33] is used to test symmetry about an unknown median. It is applied to the measured values as exemplified in Fig. 2, and it employs the bootstrap method to build the reference distribution that is then used to calculate the *p*-value, as suggested by Zheng and Gastwirth [44]. 

The symmetry test comes in two versions depending on how the *p*-value of the test is computed. In the original formulation of the test [33], the computation of the *p*-value involves a large-sample (that is, large *n*) approximation. The subsequent, modified version of the test, as developed in Ref. [44], involves bootstrap resampling [45] from a symmetrized version of the probability distribution of the data, and the reliability of such symmetrization depends on *n*.

The smallest sample size that Ref. [44] used in simulations to study the test's performance was *n* = 30. This may be taken as a hint that *n <* 30 should be considered small from the viewpoint of testing the hypothesis of symmetry, hence that the choices the Decision Tree recommends based on considerations of symmetry must be taken with a grain of salt.

### 3.1.3 Gaussian Shape

Both the Anderson-Darling [34] and Shapiro-Wilk [35] tests of Gaussian shape may reasonably be employed. The tests should be applied to the "roughly" standardized measured values: the differences between the measured values and their median, divided by the reported standard uncertainties.

The R implementation of the Anderson-Darling test, in function ad.test defined in package nortest [46], requires at least eight measured values to be available, while R function shapiro.test requires only three or more. However, these are the minimum sample sizes that render the tests practicable, yet they offer no assurances as to their power. For example, Razali and Wah [47] concluded that, in some cases and for *n* = 10, the power of these tests can be as low as 9%, and will certainly be lower still for *n <* 10.

### 3.2 Statistical Models and Data Reductions

This subsection reviews the statistical models, and corresponding data reductions, for each leaf of the Decision Tree. Implementations of the *Adaptive Weighted Average* (described in Sec. 3.2.1), of the *Hierarchical Gauss* + *Gauss* model (described in Sec. 3.2.3), and of the *Hierarchical Laplace* + *Gauss *model (described in Sec. 3.2.4), are already available in the *NIST Consensus Builder*. Figures 16 and 17, discussed in the Appendix (Sec. 6), provide computer codes that implement the *Hierarchical Skew Student *+ *Gauss* model.

#### 3.2.1 Adaptive Weighted Average

The weighted average, with weights proportional to $\left\{1/\sigma_{j}^{2}\right\}$, is the optimal KCRV when the measurement results satisfy the common mean model, *x_j_* = *µ* + *ε_j_*, where *µ* denotes the true value of the measurand, and the {*ε*_*j*_} are like outcomes of independent, Gaussian random variables with mean 0 and standard deviations {*σ*_*j*_}.

If one is prepared confidently to take *σ_j_* = *u*_c_(*x_j_*), then R function rma, with argument method="FE", defined in package metafor [20], provides the KCRV and its associated uncertainty. Otherwise, an alternative approach is required that involves treating the reported uncertainties (and possibly their numbers of degrees of freedom) as data, alongside the measured values. In either case, the DoEs require a custom treatment, properly to take the correlations into account that prevail between the measured values and the KCRV.

Considering the limitations of Cochran's *Q* test of mutual consistency, the Decision Tree suggests that the adaptive weighted average from the DerSimonian-Laird procedure [40] should be used in this case, instead of the conventional weighted average. The DerSimonian-Laird procedure includes the common weighted average as a special case, which it uses when it estimates the dark uncertainty *τ* to be 0.

The version of the DerSimonian-Laird procedure implemented in the *NIST Consensus Builder* is fully described in Ref. [18, Sec. 5.2]. This version produces the KCRV, the associated uncertainty, and the DoEs, taking into account the effective number of degrees of freedom on which the estimate of the dark uncertainty *τ* is based.

#### 3.2.2 Weighted Median

The weighted median, with weights proportional to $\left\{1/\sigma_{j}^{2}\right\}$, is the optimal KCRV when the measurement results satisfy the common mean model, *x_j_* = *µ* + *ε_j_*, where *µ* denotes the true value of the measurand, and the {*ε_j_*} are like outcomes of independent, Laplace random variables with mean 0 and standard deviations {*σ_j_*}.

The Laplace distribution, whose tails are heavier than the tails of the Gaussian distribution, accommodates measured values that deviate appreciably from the bulk of the others, yet they are deemed legitimate and are not excluded from contributing to the KCRV. This modeling device automatically dampens the influence that such extreme values have upon the KCRV and upon the uncertainty surrounding the KCRV and the DoEs.

R function weighted. median defined in package spatstat [48] serves to compute the weighted median with specified weights. Depending on the number of participants, the uncertainty associated with the KCRV, and the uncertainty component of the DoEs, are computed using either the nonparametric or the parametric version of the statistical bootstrap [45].

#### 3.2.3 Hierarchical Gauss + Gauss

The model corresponding to this leaf in the Decision Tree is the random effects model of Eq. (1), where the participants' effects, {*ℷ_j_*}, are assumed to be a sample from a Gaussian distribution with mean 0 and standard deviation *τ*, and the measurement errors {*ε_j_*} are assumed to satisfy the same assumptions as for the model underlying the weighted average.

Koepke *et al.* [18, Sec. 5.3, Sec. 6.2] described the model in detail, and the corresponding calculation of the DoEs. The procedure is implemented in the *NIST Consensus Builder*.

#### 3.2.4 Hierarchical Laplace + Gauss

The difference between this model and the *Hierarchical Gauss* + *Gauss* model described above concerns the {ℷ_j_}, which here are assumed to be a sample from a Laplace distribution with mean 0 and standard deviation *τ*, for reasons similar to those that motivate the use of the Laplace distribution in relation with the weighted median [49, Sec. 5.1.5]. The corresponding procedure is implemented in the *NIST Consensus Builder*. Rukhin and Possolo [50] propose a similar model, except that the participants specific measurement errors, {*ε*_*j*_} in Eq. (1), are assumed to have Laplace, rather than Gaussian distributions.

#### 3.2.5 Hierarchical Skew Student + Gauss

The *Hierarchical Skew Student* + *Gauss* model describes the measured values according to Eq. (1) assuming that the participants' effects, {*ℷ_j_*}, are a sample from a skew-*t* distribution [51], as introduced by Koepke and Possolo [52] in the context of interlaboratory studies and meta-analysis. The Appendix (Sec. 6) provides details about this model and presents stand-alone Stan and R computer codes that implement it.

The *Hierarchical Skew Student* + *Gauss* model, which is used for the results of the KC discussed in Sec. 4.5, has adaptive tail-heaviness controlled by a non-negative parameter *v* (number of degrees of freedom), and also adaptive asymmetry quantified by a real-valued, skewness parameter *α* (with *α >* 0 indicating skewness to the right). Thus, the *Hierarchical Skew Student* + *Gauss* model accommodates sets of measurement results that arrange themselves asymmetrically relative to the consensus value, and that may possibly include measured values that deviate considerably from the bulk of the others, yet are deemed legitimate and are not excluded from contributing to the KCRV.

## 4 Examples

The first five examples are from past KCs organized by the CCQM-IAWG, and serve to demonstrate the five leaves of the Decision Tree. The other examples, involving measurements of radionuclides and of microwave power, serve to show the wide applicability of the Decision Tree as a guide to model selection. The acronyms and initialisms used in these examples, to denote either participants in KCs or individual studies in interlaboratory studies, are defined in the published final reports or referenced articles.

Section 4.1 illustrates the selection and results corresponding to the adaptive weighted average as applied to the estimation of the KCRV for the mass fraction of tin in tomato paste in CCQM-K45.

Section 4.2 uses measurement results for the mass fraction of zinc in bovine liver, from CCQM-K145, for which the Decision Tree recommends the weighted median.

Section 4.3 employs to the *Hierarchical Gauss* + *Gauss* procedure to reduce the measurement results for the mass fraction of nickel in bovine liver, also from CCQM-K145.

Section 4.4 addresses a KC with apparently outlying yet substantively acceptable measurement results for the mass fraction of lead in lead-free solder, from CCQM-K88, for which the *Hierarchical Laplace* + *Gauss* model seems to be the best among those considered in the Decision Tree.

Section 4.5 offers an instance of application of the *Hierarchical Skew Student* + *Gauss* procedure to reduce measurement results for the mass fraction of lead in wine, from CCQM-K30.1.

Section 4.6 suggests that the Decision Tree is a flexible, general purpose replacement even for *ad hoc* procedures such as the power-moderated weighted mean proposed by Pommé and Keightley [56], which has recently been adopted by Section II (Measurement of radionuclides) of the Consultative Committee for Ionizing Radiation (CCRI), or the procedure that Ref. [57] proposed for dealing with discrepant data.

Finally, Sec. 4.7 briefly reviews the recommendation of the Decision Tree for a particular set of measurement results from CCEM.RF-K25.W, two of which the Consultative Committee for Electricity and Magnetism (CCEM) chose to set aside because they were "statistical outliers."

In all cases, the goal is not to offer an alternative KCRV, associated uncertainty, or DoEs, but to illustrate how the Decision Tree may be used to arrive at a reasonable candidate solution for how to reduce the data from a KC.

### 4.1 CCQM-K45 Tin in Tomato Paste

The final report of CCQM-K45 [36] explains that UME deviated significantly from the protocol, and for this reason their result was excluded from the calculation of the KCRV. The KCRV was the simple average of the values measured by the other four participants, 227.1 mg*/*kg, and the associated standard uncertainty was 1.2 mg*/*kg. For this illustration, we will set UME's result aside.

The final report evaluated the uncertainty associated with the KCRV as the sample standard deviation of the measured values divided by the square root of 4, which is the GUM's Type A evaluation of uncertainty for the simple average of replicated observations made under conditions of repeatability. The reported uncertainties were disregarded both in the calculation of the KCRV and in the evaluation of the associated uncertainty.

The final report appears to recognize implicitly that a weighted average might have been preferable (the largest reported uncertainty was 4.3 times larger than the smallest reported uncertainty), but it points out that (1) "KRISS used a coverage factor equal to 2.45, which indicates only 6 degrees of freedom. As a result, the weighted mean is not considered the best estimate of the KCRV, as all results do not have sufficient degrees of freedom," and then it adds that (2) "as only 4 values can be used to calculate the KCRV, the median is also not considered the best estimate of the KCRV" [36, p. 5].

Table 1 lists the mass fraction of tin in tomato paste for CCQM-K45, the associated expanded uncertainties reported by the participants, and the corresponding coverage factors. The fact that KRISS did use a coverage factor other than two is no obstacle for the procedures described in the Decision Tree because they can handle finite numbers of degrees of freedom. KRISS's coverage factor corresponds to six effective degrees of freedom.

**Table 1:** CCQM-K45, Mass Fraction of Tin in Tomato Paste: measurement results and coverage factors for the expanded uncertainties for 95% coverage. The acronyms and initialisms listed under LAB are defined in Ref. [36].

LAB	*w /* mg*/*kg	*U* (*w*) */* mg*/*kg	*k*
KRISS	226.26	1.94	2.45
LGC	224.6	3.0	2
LNE	230.42	5.97	2
PTB	227.0	1.4	2
UME	209.8	7.6	2

As illustrated in Fig. 2, and stated in its caption, Cochran's *Q* test, the symmetry test, and the test of Gaussian shape, did not reject the corresponding hypotheses under test. In these circumstances, the Decision Tree recommends the adaptive weighted average for the KCRV, which turned out to be 226.5 mg*/*kg.

Even though Cochran's test yielded a *p*-value of 0.27, the DerSimonian-Laird estimate of *τ*, 0.601 mg*/*kg, is not zero. Thus, *τ* = 0.601 mg*/*kg will be used to modulate the weights of the weighted average as described in Ref. [18] and implemented in the *NIST Consensus Builder*.

The standard uncertainty associated with the KCRV of 0.705 mg*/*kg, evaluated using the parametric statistical bootstrap, recognizes the six degrees of freedom supporting KRISS's reported uncertainty. This evaluation is larger than the "internal" evaluation, which uses the formula $1/\sqrt{\Sigma_{j=1}^{n}(1/u_{c}(w_{j}}))$ (with *n* = 4 because UME's result was not included in the calculation of the KCRV), and yields 0.5 mg*/*kg.

**Fig. 3:**
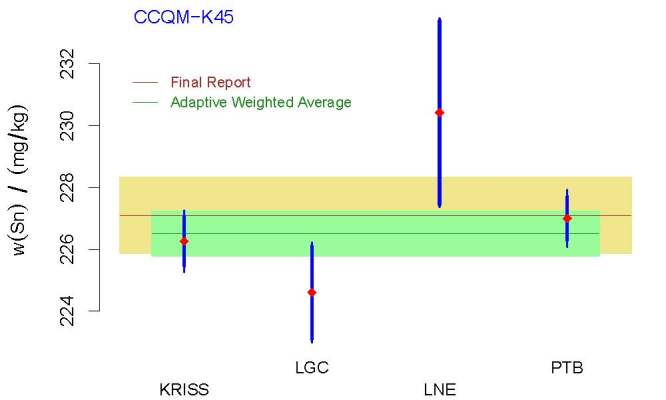
Measurement results from CCQM-K45 that were used to determine the KCRV and evaluate the associated uncertainty. The acronyms and initialisms designating the participants are defined in Ref. [36]. The red diamonds represent the measured values, and the vertical, thick blue line segments represent *±*1 standard uncertainty intervals around the measured values. The minute, thin extensions of these vertical line segments reflect the contribution from dark uncertainty. The horizontal, dark green line indicates the KCRV produced by the approach recommended by the Decision Tree and the pale green band depicts the associated standard uncertainty. The horizontal, brown line and khaki band indicate the results from the final report. The two versions of the KCRV are not significantly different, but the KCRV recommended by the Decision Tree has appreciably smaller uncertainty than its counterpart from the final report.

**Fig. 4:**
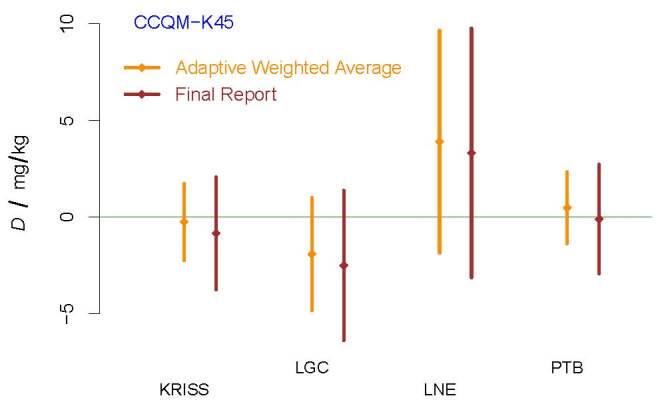
DoEs for the participants in CCQM-K45, excluding UME. The acronyms and initialisms designating the participants are defined in Ref. [36].

Figure 3 depicts and compares the results for the KCRV, as per the final report, and their counterparts from the Decision Tree. The expanded uncertainty components of the DoEs were evaluated using the parametric bootstrap and recognize the small number of degrees of freedom supporting the standard uncertainty reported by KRISS. Figure 4 depicts the two versions of the DoEs.

### 4.2 CCQM-K145 Zinc in Bovine Liver

The final report of CCQM-K145 [58], regarding the mass fraction of zinc, set aside results from INRAP, UNIIM, KEBS, INRIM, and EXHM for the reasons explained in the final report. The result from VNIIFTRI was also set aside without an explicit explanation, but presumably owing to this participant having used a commercial reference material as calibrant for the determination of the mass fraction of zinc [58, Table 5]. Table 2 lists the measurement results and indicates those that the final report included in the calculation of the KCRV.

The final report uses the median as KCRV. However, use of the median rendered the preliminary data selection exercise pointless, whereby "suspected extreme results" were investigated and set aside, because the median of all the measured values is identical to the median of the selected values, 456.2 mg*/*kg. This is no coincidence: the median is resistant to up to 50% of the measured values being "extreme results." The associated uncertainty likely will vary depending on which results are set aside.

**Table 2:** CCQM-K145, Mass Fraction of Zinc in Bovine Liver: measurement results, coverage factors for the expanded uncertainties for 95% coverage, and whether they were included or not in the calculation of the KCRV. The acronyms and initialisms listed under LAB are defined in Ref. [58].

LAB	*w*	*u*(*w*)	*U* (*w*)	*k*	KCRV		
* /* mg*/*kg							
EXHM	420.6	11.5	23.0	2	NO		
INRAP	429.71	9.48	18.96	2	NO		
UNIIM	436	9	18	2	NO		
LATU	453	5.6	11	2	YES		
INACAL	453.1	8.8	17.6	2	YES		
GLHK	453.6	7.7	15.4	2	YES		
NMIA	454.1	4.2	8.6	2.04	YES		
NIM	454.5	5.5	11	2	YES		
KRISS	454.5	6.7	13.2	1.96	YES		
LGC	454.5	3.9	7.8	2	YES		
NMISA	454.5	6.1	12.2	2	YES		
JSI	455	14	28	2	YES		
NIST	456.2	2.0	4.0	2.04	YES		
UME	457	4	7	2	YES		
HSA	459	7.1	14	2	YES		
PTB	459.4	1.7	3.4	2.03	YES		
RISE	460.5	3.2	6.3	2	YES		
SYKE	460.9	11.5	23.0	2	YES		
INMC	461	6.5	13	2	YES		
NRC	462	5	10	2	YES		
NIMT	462	13.2	27	2	YES		
NMIJ	462	3	6	2	YES		
KEBS	474.22	16.46	38.92	2.36	NO		
INRIM	491.7	10.0	20.1	2	NO		
VNIIFTRI	524	16	32	2	NO		

The main issues with the data reduction in the final report are these:

⏺ The median ignores the reported uncertainties, which are an integral part of the measurement results.
⏺ Equation (3) in the final report, which was used to evaluate the standard uncertainty for the median, in fact assumes that the measured values are a sample from a Gaussian distribution. In this case, Eq. (3) produces a significant undervaluation of the uncertainty for the KCRV.
⏺ The calculation of expanded uncertainties for the DoEs using Eq. (5) of the final report is incorrect for two reasons: (1) it ignores correlations between the KCRV and the measured values; and (2) the evaluation of *u*(KCRV) uses a formula that assumes that the data are a sample from a Gaussian distribution, and even then only approximately when the number of participants is large.

**Fig. 5:**
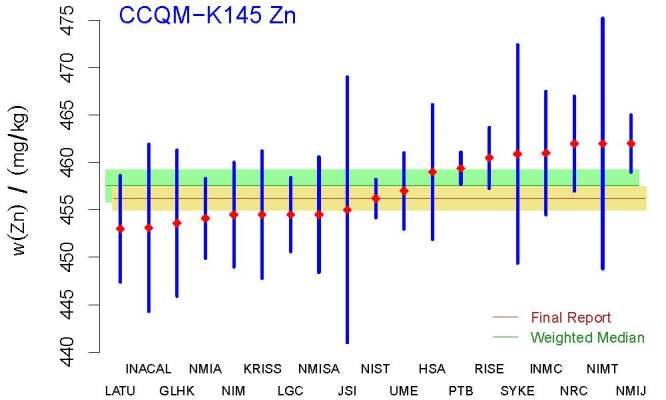
Selected measurement results for the mass fraction of zinc in CCQM-K145 that contributed to the calculation of the KCRV. The acronyms and initialisms designating the participants are defined in Ref. [58]. The red diamonds represent the measured values, and the vertical, blue line segments represent *±*1 standard uncertainty intervals around the measured values. Even though the median and the weighted median are not significantly different, the final report likely under-evaluated the uncertainty associated with the median.

Figure 5 depicts the selected measured values (red diamonds) and the *±*1 standard uncertainty intervals around the measured values (blue segments). Both the Shapiro-Wilk and Anderson-Darling tests reject the hypothesis that the measured values are a sample from a Gaussian distribution (with *p*-values less than, even if close to 1%), which invalidates the evaluation of standard uncertainty for the KCRV in the final report. The measurement results are mutually consistent as judged by Cochran's (chi-squared) *Q* test (*p*-value of 0.96).

The Decision Tree, considering that the results appear to be homogeneous but not Gaussian, suggests the weighted median for the KCRV, with weights inversely proportional to the squared reported uncertainties. Applying R function weighted .median, defined in package spatstat (version 1.64-1) [48], yields 457.55 mg*/*kg (bias-corrected by the statistical bootstrap).

**Fig. 6:**
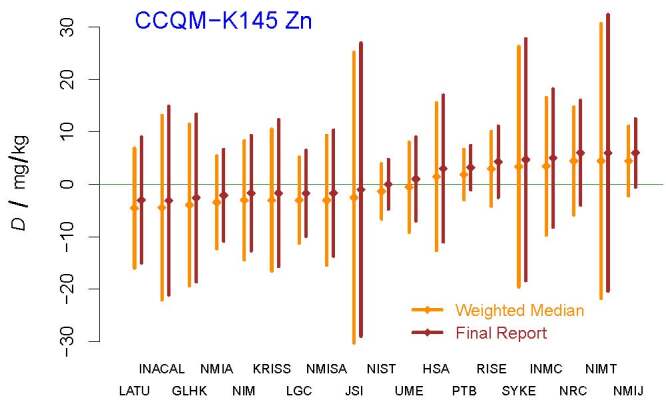
DoEs for the participants in CCQM-K145 whose measurement results were included in the calculation of the KCRV for the mass fraction of zinc. The acronyms and initialisms designating the participants are defined in Ref. [58].

The standard uncertainty of the weighted median, 1.65 mg*/*kg, which was computed using the nonparametric statistical bootstrap [45], is approximately 1.4 times larger than the corresponding value in Table 20 of the final report. The Monte Carlo method was used to produce evaluations of the DoEs, {*D*_*j*_*±U*_95%_(*D*_*j*_)}, which are depicted in Fig. 6.

### 4.3 CCQM-K145 Nickel in Bovine Liver

In the final report of CCQM-K145 [58], the KCRV is the median of a selected subset of the measured values, with standard uncertainty computed according to Eq. (3) of the final report. The DoEs were computed according to Equations (4)-(5). The results from UNIIM, NMISA, NIMT, KEBS, and EXHM were set aside for the reasons explained in the final report. The result from VNIIFTRI was also set aside likely for the same reason mentioned in Sec. 4.2. Table 3 lists the measurement results and indicates those that were included in the calculation of the KCRV, as per the final report.

The main issues with the original analysis presented in the Final Report are the following:

⏺ The median ignores the reported uncertainties, which are an integral part of the measurement results. The median also is blind to *dark uncertainty* [19], the excess dispersion of the measurement results that renders them mutually inconsistent.
⏺ The calculation of expanded uncertainties for the DoEs using Eq. (5) of the final report is incorrect for two reasons: (1) it ignores correlations between the KCRV and the measured values; and (2) the evaluation of *u*(KCRV) uses a formula that assumes that the data are a sample from a Gaussian distribution, and even then only approximately when the number of participants is large.

The measurement results are not mutually consistent as judged by Cochran's (chi-squared) *Q* test (*p*-value 0.0007). The measured values may reasonably be regarded as a sample from a symmetrical distribution according to the statistical test recommended in Ref. [33], which is implemented in R package symmetry [59].

Additionally, the roughly standardized measured values, {(*x_j_ - m)/u(x_j_*)}, where *m* is the median of the measured values, may be reasonably regarded as a sample from a Gaussian distribution, according to both the Shapiro-Wilk and Anderson-Darling tests. Therefore the recommended analysis is the *Hierarchical Gauss* + *Gauss* procedure, which is already implemented in the *NIST Consensus Builder*.

**Fig. 7:**
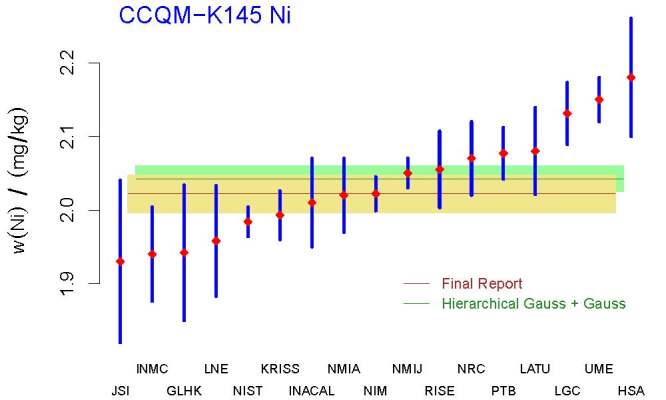
Selected measured values (red diamonds), and the *±*1 standard uncertainty intervals around the measured values (blue segments), for the mass fraction of nickel in CCQM-K145. The acronyms and initialisms designating the participants are defined in Ref. [58]. Median (brown horizontal line) and associated standard uncertainty (half the height of the khaki band) are shown according to the final report, and their counterparts for the KCRV (labeled Hierarchical Gauss + Gauss) recommended by the Decision Tree.

**Fig. 8:**
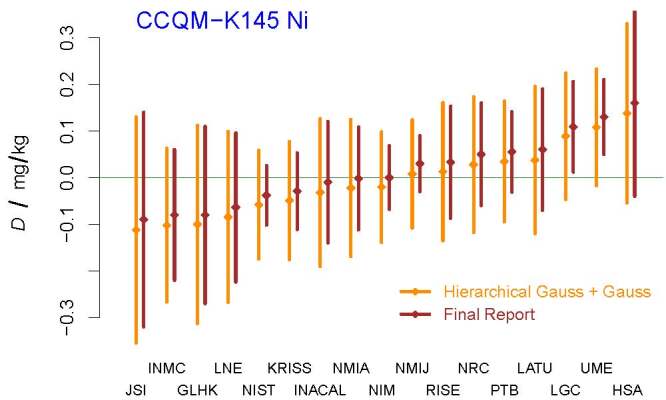
DoEs corresponding to the determinations of the mass fraction of nickel in CCQM-K145. The acronyms and initialisms designating the participants are defined in Ref. [58]. Note the difference in the results for LGC and UME corresponding to the *Hierarchical Gauss* + *Gauss* procedure and to the final report.

The resulting KCRV, labeled Hierarchical Gauss + Gauss in Fig. 7, is 2.042 mg*/*kg. The associated standard uncertainty equals 0.017 mg*/*kg, which is larger than the corresponding value in Table 20 of the final report. The DoEs depicted in Fig. 8 also were produced by the *NIST Consensus Builder*. Note that these DoEs suggest different conclusions for LGC and UME than the final report.

**Table 3:** CCQM-K145, Mass Fraction of Nickel in Bovine Liver: measurement results, coverage factors for the expanded uncertainties for 95% coverage, and whether they were included or not in the calculation of the KCRV. The acronyms and initialisms listed under LAB are defined in Ref. [58].

LAB	*w*	*u*(*w*)	*U* (*w*)	*k*	KCRV
					
		*/* mg*/*kg			
UNIIM	0.147	0.016	0.033	2	NO
NMISA	0.902	0.038	0.076	2	NO
EXHM	1.017	0.05	0.100	2	NO
NIMT	1.65	0.06	0.13	2	NO
JSI	1.93	0.11	0.22	2	YES
INMC	1.94	0.06387	0.13	2	YES
GLHK	1.942	0.092	0.183	2	YES
LNE	1.958	0.075	0.15	2	YES
VNIIFTRI	1.96	0.09	0.18	2	NO
NIST	1.984	0.020	0.047	2.31	YES
KRISS	1.993	0.033	0.067	2.06	YES
INACAL	2.01	0.06	0.13	2	YES
NMIA	2.02	0.05	0.1	2.02	YES
NIM	2.022	0.023	0.046	2	YES
NMIJ	2.05	0.02	0.04	2	YES
RISE	2.055	0.052	0.10	2	YES
NRC	2.07	0.05	0.10	2	YES
PTB	2.077	0.035	0.071	2.00	YES
LATU	2.08	0.059	0.12	2	YES
LGC	2.131	0.042	0.084	2	YES
UME	2.15	0.03	0.06	2	YES
HSA	2.18	0.08	0.15	2	YES
KEBS	4.63	0.94	2.22	2.36	NO

### 4.4 CCQM-K88 Lead in Lead-Free Solder

Even though participants in CCQM-K88 [60] were allowed to use any suitable method of measurement, at its meeting of April 11-12, 2011, the CCQM-IAWG decided that the KCRV would be estimated based on the measurement results obtained using either inductively coupled plasma mass spectrometry (ID-ICP-MS) (four participants) or isotope dilution thermal ionization mass spectrometry (ID-TIMS) (one participant), therefore excluding the results from the other five participants. The reported KCRV was the median, 197.2 mg*/*kg, with expanded uncertainty of 0.9 mg*/*kg for 95% coverage.

The same as in the other examples in this collection, the purpose of this review is not to suggest an alternative to the KCRV that was adopted originally, or to its uncertainty or to the DoEs, but instead to use the measurement results from all participants, listed in Table 4, to illustrate how a set of results, including some that deviate markedly from the bulk of the others, may be reduced when there is no reason to set any of them aside.

**Table 4:** CCQM-K88, Mass Fraction of Lead in Lead-Free Solder: measurement results, and whether the final report included them or not in the calculation of the KCRV. The acronyms and initialisms listed under LAB are defined in Ref. [60].

LAB	*w /* mg*/*kg	*U*(*w*) */* mg*/*kg	*k*	KCRV
INMETRO	179	4	2	NO
VNIIM	194.2	10	2	NO
NIM	195.8	2.6	2	YES
NMIJ	196.7	1.52	2	YES
KRISS	197.2	2	2	YES
PTB	197.9	1.9	2	YES
BAM	198.29	0.5	2	YES
INTI	199	4	2	NO
NIST	199.43	0.7	2	NO
NRC	202.4	18.6	2	NO

All 10 measurement results were selected for this exercise. The measurement results are mutually inconsistent as judged by Cochran's *Q* (chi-squared) test, which yields a *p*-value of less than 0.0001. Furthermore, the dispersion of the measured values, as gauged using R function mad, is about two times larger than the median of the reported standard uncertainties.

**Fig. 9:**
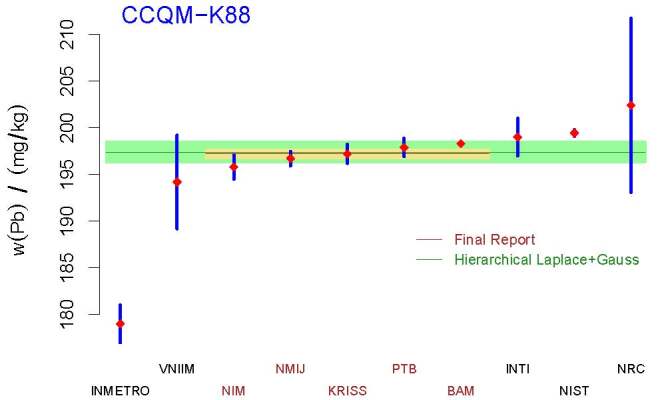
Measurement results for the mass fraction of lead in CCQM-K88, and comparison of the KCRV adopted in the final report (horizontal brown line with associated standard uncertainty represented by the khaki band), which included only the values corresponding to the participants labeled in brown, with the KCRV recommended by the Decision Tree (horizontal, dark green line), 197.4 mg*/*kg. The pale green band depicts the associated standard uncertainty, 1.1 mg*/*kg. The dark green and brown lines are almost indistinguishable. The acronyms and initialisms designating the participants are defined in Ref. [60].

**Fig. 10:**
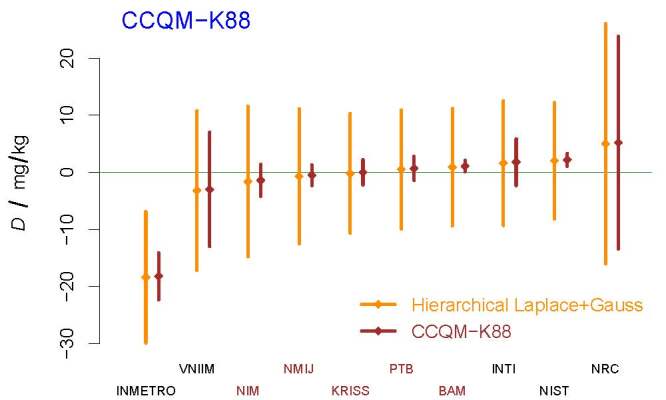
The DoEs listed in the final report fail to take into account the substantial, prevailing component of dark uncertainty, unduly penalizing BAM and NIST. The acronyms and initialisms designating the participants are defined in Ref. [60].

The Anderson-Darling test of Gaussian shape, applied to the roughly standardized measured values, with a *p*-value of 0.03, suggests that the Gaussian model is not appropriate for these data. But since the test of symmetry proposed in Ref. [33] yields a *p*-value of 0.22, the Decision Tree recommends the *Hierarchical Laplace* + *Gauss* model, with results depicted in Fig. 9. The DoEs, {*D_j_±U*_95%_(*D_j_*)}, were computed as explained in [18, Sec. 6.2]. The DoEs depicted in dark orange (leftmost of each pair) in Fig. 10 are from the present study, appreciable difference between the corresponding expanded uncertainties is attributable to the dark uncertainty, which is substantial ($\hat{\tau }$ = 4.3 mg*/*kg) when none of the measurement results is set aside. This is reflected in the DoEs corresponding to the *Hierarchical Laplace* + *Gauss* model.

### 4.5 CCQM-K30.1 Lead in Wine

The final report for CCQM-K30.1 [61] explains that only the results for the five laboratories that used isotope dilution mass spectrometry (IDMS) as measurement method were used to calculate the KCRV and associated uncertainty. This left five other results out of this calculation. The KCRV, 12.12 ng*/*g, was the median of the five selected measured values, and the associated standard uncertainty, 0.03 ng*/*g, was computed using the formula listed on the last line of page 7 of the final report.

This formula is inappropriate because it applies only to data from a Gaussian distribution, and then only approximately when the number of participants is large, which is not the case here. The calculation of the expanded uncertainty portion of the DoEs also was done incorrectly, using the formula on page 8 of the final report, because it neglects the correlation between the KCRV and the measured values that were used to compute the KCRV.

**Fig. 11:**
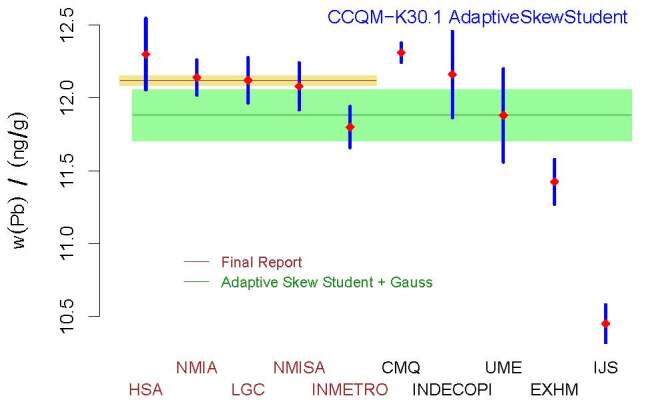
CCQM-K30.1, Lead in Wine: measured values (red diamonds) of the mass fraction of lead and associated standard uncertainties, where the vertical, blue line segments represent the {*w_j_* ± *u(w_j_*)}; KCRV (horizontal, short, brown line) and associated standard uncertainty (khaki band) from the Final Report, determined by the measurement results from HSA, NMIA, LGC, NMISA, and INMETRO only; and KCRV recommended by the Decision Tree (horizontal, long, dark green line), 11.88 ng*/*g, with uncertainty band representing *±*1 standard uncertainty, 0.17 ng*/*g, obtained using the measurement results from all 10 participants. The acronyms and initialisms designating the participants are defined in Ref. [61].

**Fig. 12:**
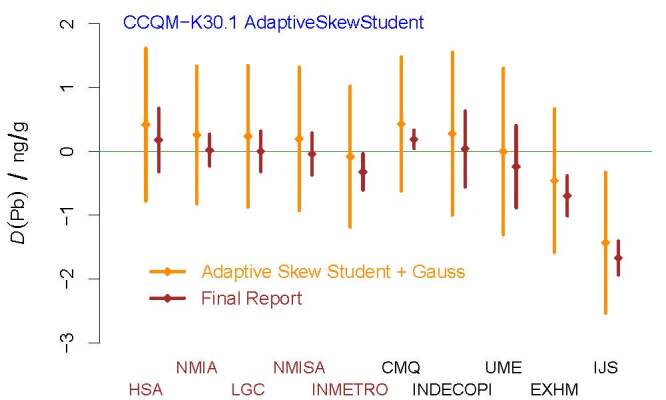
DoEs corresponding to the mass fraction of lead in wine, for all the participants in CCQM-K30.1, produced as recommended by the Decision Tree. The DoEs depicted in dark orange (leftmost of each pair) are from this reevaluation, and the DoE depicted in brown (rightmost in each pair) are from the final report. The acronyms and initialisms designating the participants are defined in Ref. [61].

Table 5 lists the measurement results used for this illustration, which serves to show how a set of results where the measured values exhibit marked asymmetry may be reduced when there is no reason to set any of them aside. The measurement results were reordered so that those pertaining to the five laboratories selected for calculation of the KCRV in the final report appear on the left-hand side of Figures 11 and 12.

**Table 5:** CCQM-K30.1, Lead in Wine: measurement results, and coverage factors for 95% coverage. The acronyms and initialisms listed under LAB are defined in Ref. [61].

LAB	*w /* mg*/*kg	*u*(*w*) */* mg*/*kg	*U* (*w*) */* mg*/*kg	*k*
HSA	12.3	0.25	0.49	2
NMIA	12.14	0.12	0.24	2.03
LGC	12.12	0.155	0.31	2
NMISA	12.08	0.16	0.32	2
INMETRO	11.8	0.14	0.28	2
CMQ	12.31	0.06	0.13	2
INDECOPI	12.16	0.3	0.59	2
UME	11.88	0.32	0.64	2
EXHM	11.424	0.153	0.306	2
IJS	10.45	0.13	0.26	2

All 10 measurement results were selected for this exercise. The measurement results are mutually inconsistent as judged by Cochran's *Q* (chi-squared) test, which yields a *p*-value smaller than 0.0001, and the dispersion of the measured values is about twice as large as the median of the reported standard uncertainties. The symmetry test suggested in Ref. [33] yields a *p*-value of 0.007, suggesting significant asymmetry.

In these circumstances, the Decision Tree recommends the *Hierarchical Skew Student* + *Gauss* model described in Sec. 3.2.5. The corresponding estimate of the KCRV is 11.88 ng*/*g, with standard uncertainty of 0.17 ng*/*g. The dark uncertainty, *τ*, has a posterior mean of 0.5 ng*/*g and posterior standard deviation of 0.2 ng*/*g. The DoEs, {*D*_*j*_*±U*_*95%*_(*D*_*j*_)}, were computed as explained in Ref. [18, Sec. 6.2], and are depicted in Fig. 12.

The asymmetry parameter, *α*, had posterior mean *-*4 and posterior standard deviation approximately 3, thus capturing the long left tail of the measured values apparent in Fig. 11. The number of degrees of freedom, *v*, of the underlying skew-*t* distribution, had posterior mean of 11, posterior standard deviation of 6, and posterior median of 9, approximately.

### 4.6 Measurements of Radionuclides

#### 4.6.1 Equivalent Activity of ^65^Zn

Table 6 lists the measurement results for the equivalent activity of ^65^Zn obtained in linked KCs BIPM.RI(II)-K1.Zn-65 and CCRI(II)-K2.Zn-65, using the SIR (International Reference System) at the BIPM (International Bureau of Weights and Measures, Sèvres, France) [62, Tables 4a+4b].

**Table 6:** Measurement results for the equivalent activity of ^65^Zn obtained in KCs BIPM.RI(II)-K1.Zn-65 + CCRI(II)-K2.Zn-65, using the SIR (International Reference System) at the BIPM (International Bureau of Weights and Measures, Sèvres, France) [62, Tables 4a+4b], with the values measured by ASMW and BARC rounded as per the corresponding *KCRV File* (C. Michotte, personal communication, January 4, 2021). The results from the NMIs in boldface (BEV, ENEA, and SMU)) were not included in the calculation of the KCRV. The acronyms and initialisms listed under LAB are defined in Ref. 62.

LAB	*A*_e_(^65^Zn)		*u*(*A*_e_(^65^Zn)) LAB				*A*_e_(^65^Zn)	*u*(*A*_e_(^65^Zn))
			*/*kBq			*/*kBq	
ANSTO	29610		100			KRISS	29780	130
ASMW	29480		130			LNE-LNHB	29810	130
NIST	29840		190			LNMRI/IRD	30040	160
BARC	29130		310			MKEH	29590	120
BEV	29670		330			NMIJ	29700	150
CMI-IIR	29850		170			NMISA	29870	110
CNEA	30030		130			NPL	29990	110
ENEA	29660		120			PTB	29710	130
IFIN-HH	29550		150			SMU	29200	670
IRA	29720		140			VNIIM	29727	87
IRMM	29661		68					
							

The final report explains that "In May 2013 the CCRI(II) decided to no longer calculate the key comparison reference value (KCRV) by using an unweighted mean but rather by using the power-moderated weighted mean" [56]. Its authors suggest that this procedure "can be regarded as an upgrade of the well-established Mandel-Paule (M-P) mean" [63, 64].

**Fig. 13:**
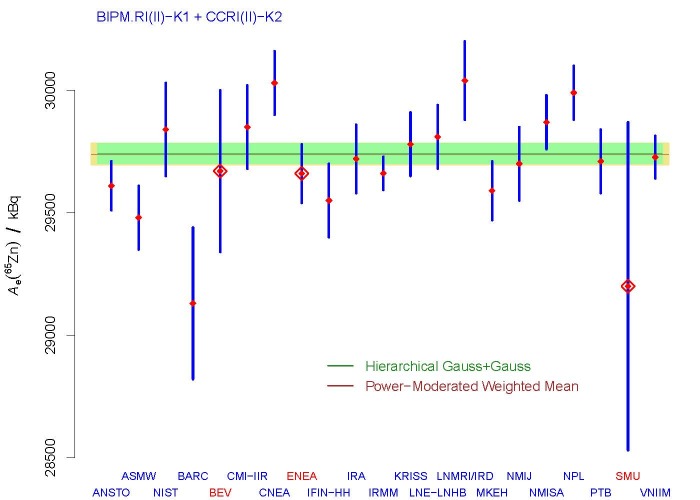
BIPM.RI(II)-K1.Zn-65 + CCRI(II)-K2.Zn-65: measured values (small, solid, red diamonds) of the equivalent activity of ^65^Zn obtained using the SIR (International Reference System) at the BIPM (International Bureau of Weights and Measures, Sèvres, France), and associated, reported standard uncertainties, where the vertical, blue line segments represent the {*A*_*e*_(^65^Zn)±*u*(*A*_*e*_(^65^Zn))}; KCRV (29740 kBq, horizontal, brown line) and associated standard uncertainty (43 kBq, khaki band) from the final report [62], determined by the measurement results from ANSTO, ASMW, NIST, BARC, CMI-IIR, CNEA, IFIN-HH, IRA, IRMM, KRISS, LNE-LNHB, LNMRI/IRD, MKEH, NMIJ, NMISA, NPL, PTB, and VNIIM only (Ref. [62] explains the meaning of these acronyms and initialisms); and corresponding, alternative KCRV recommended by the Decision Tree (29742 kBq, horizontal, dark green line), with uncertainty band representing *±*1 standard uncertainty (40 kBq, pale green band) obtained using the measurement results from the same selected participants. The large, open, red diamonds indicate the three participants whose results the CCRI(II) chose to exclude from the computation of the KCRV because the standardization procedure used for them did not rely on a primary method.

Considering only the same results that the CCRI(II) chose to include in the calculation of the KCRV, the Decision Tree suggests the *Hierarchical Gauss* + *Gauss* model for these data. The KCRV produced by this model, 29742 kBq *±* 40 kBq, obtained using the *NIST Consensus Builder*, is statistically indistinguishable from its counterpart listed in the final report, 29740 kBq *±* 43 kBq. The associated uncertainties, too, are almost identical (Fig. 13).

However, there are notable differences between the DoEs corresponding to these two approaches: the expanded uncertainty components of the DoEs from the *Hierarchical Gauss* + *Gauss* model are appreciably larger than those from the power-moderated weighted mean, except for SMU, which is one of the NMIs whose result was not included in the calculation of the KCRV. These differences are particularly consequential for CNEA (Comisión Nacional de Energía Atómica, Argentina) and NPL (National Physical Laboratory, United Kingdom).

The reason for this persistent difference is the fact that the *NIST Consensus Builder* heeds the advice in Ref. [18], to the effect that the {*U*_95%_(*D*_*j*_)} should be evaluated consistently with their primary goal of identifying participants with "unusual" results, in the sense that their measured values lie "beyond the range allowed by the model", as suggested by Jones and Spiegelhalter [22].

**Fig. 14:**
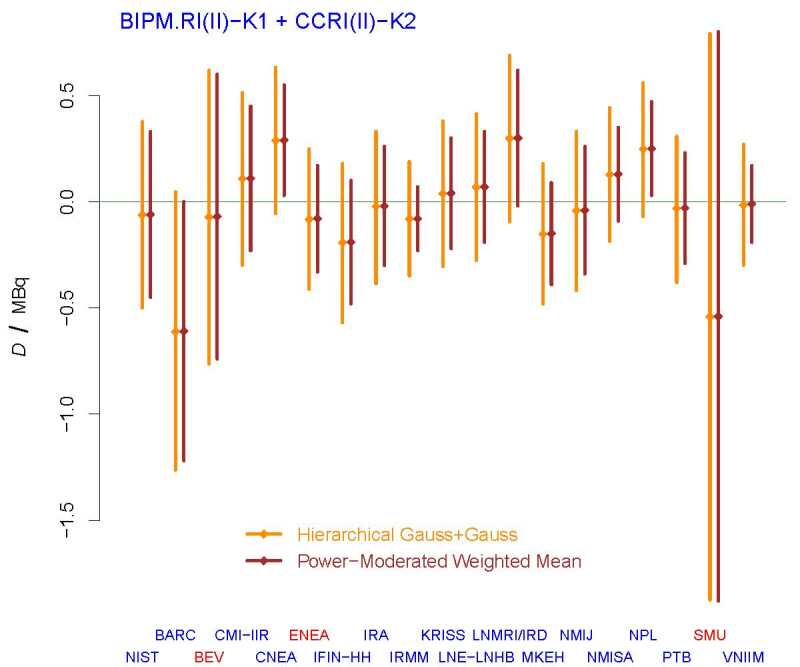
DoEs for selected participants in BIPM.RI(II)-K1.Zn-65 + CCRI(II)-K2.Zn-65. The diamonds represent the differences, {*D*_*j*_}, between measured values and the KCRV, and the vertical line segments represent {*D*__*j*__*±U*_95%_(*D*__*j*__ )}, in brown for the DoEs listed in the final report [62, Table 5], and in dark orange for the *Hierarchical Gauss* + *Gauss* model recommended by the Decision Tree. The final report does not report DoEs for ANSTO or ASMW because their measurement results were already more than 20 years old when the comparison was made (C. Michotte, personal communication, January 4, 2021). The DoE for NIST is based on the measurement result from 2001, while the result from 1999 was used in the calculation of the KCRV because it was the closest in time to the relevant calibration (C. Michotte, personal communication, January 4, 2021). Other than for SMU, the expanded uncertainties produced by the latter model are larger than those listed in the final report: these differences are particularly consequential for CNEA (Comisión Nacional de Energía Atómica, Argentina) and NPL (National Physical Laboratory, United Kingdom). The acronyms and initialisms designating the participants are defined in Ref. [62]. Those that appear in red indicate the participants whose measurement results were excluded from the computation of the KCRV.

Determining whether participants have "unusual" results involves consideration of the estimate of dark uncertainty, $\hat{\tau }$ = 96 kBq, which in this case amounts to about 74% of the median of the reported standard uncertainties. The differences between the uncertainties in the DoEs corresponding to the original treatment and to the *Hierarchical Gauss* + *Gauss* model depicted in Fig. 14, suggest that the power-moderated weighted mean may not be propagating dark uncertainty to the DoEs properly.

A measurement result from P3KRBiN (Research Center for Radiation Safety and Nuclear Biomedics, Indonesia) obtained in 1993, 28540 kBq *±* 89 kBq, had originally been considered for inclusion in the KCRV, but was later excluded because the power-moderated weighted mean suggested that it was an outlier (C. Michotte, personal communication, January 26, 2021).

If this result is placed alongside those that contributed to the determination of the KCRV, then the Decision Tree recommends the *Hierarchical Laplace* + *Gauss* model, and in the process secures protection against the influence of this markedly discrepant result, yielding a KCRV that is statistically indistinguishable from those listed above: 29719 kBq *±* 56 kBq.

#### 4.6.2 Half-Life of ^90^Sr

Koepke *et al.* [18, Sec. 7.2] consider a set of measurement results for the half-life of ^90^Sr that MacMahon *et al.* [57] used to illustrate a procedure they propose to compute a consensus value in situations where there are "discrepant data" in the sense that the results are heterogeneous.

The Decision Tree recommends the *Hierarchical Skew Student* + *Gauss* model for these data, which are listed in Table 7, because Cochran's test yields a *p*-value less than 0.0001, both the Shapiro-Wilk and Anderson-Darling tests yield *p*-values less than 0.0003, and the symmetry test yields *p*-value of 0.02.

**Table 7:** Measured values of the half-life of ^90^Sr and associated standard measurement uncertainty, expressed in days, reported in Ref. [57]. The labels listed under STUDY correspond to the references in Ref. [57, Table 1].

STUDY	*T*_1/2_(^90^Sr)*/*d	*u*(*T*_1/2_(^90^Sr))*/*d
WT55	10120	150
An58	10700	580
Fl65	10230	150
Fl65	10410	330
Ho77	10636	88
La78	10282	12
Ra83	10588	91
Ko89	10665	37
Ma94	10561	14
WL96	10495	4
Sc04	10557	11

MacMahon *et al.* [57] conclude that the "best" estimate of the half-life of ^90^Sr, expressed in years, is 10551 d (28.9 years), with standard uncertainty 14 d. Their counterparts based on the choice recommended by the Decision Tree are 10494 d (28.7 years) and 41 d respectively. Therefore, these two estimates of the half-life are not significantly different, yet the latter was produced using a general purpose, model-based procedure, while the former is the result of a tailor-made, *ad hoc* recipe.

### 4.7 CCEM.RF-K25.W Thermistor Power Sensor Efficiency

The CCEM key comparison CCEM.RF-K25.W [65] involved the measurement of the effective efficiency of two commercial, waveguide, temperature-compensated thermistor power sensors by several NMIs. The effective efficiency, *η*_EFF_, is the ratio of the substituted DC power to the total absorbed radio-frequency power. Table 8 lists the results of measurements made at 36 GHz using traveling standard PTB-1.

**Table 8:** Measurement results for the PTB-1 power sensor, at 36 GHz, from CCEM.RF-K25.W [65, Table 5]. The final report excluded the measurement results from NIM and NRC from the calculation of the KCRV because they were deemed to be "statistical outliers." The acronyms and initialisms listed under LAB are defined in Ref. [65].

LAB	*η*_EFF_	*u*(*η*_EFF_)		
KRISS	0.9143	0.0104		
LNE	0.9157	0.0018		
NIST	0.9184	0.0064		
NPL	0.9167	0.0060		
PTB	0.9153	0.0031		
VNIIFTRI	0.9160	0.0079		
NIM	0.8360		0.0072
NRC	0.9375		0.0130

The measurement results from NIM and from NRC were left out of the calculation of the KCRV because they were deemed to be "statistical outliers." The estimate of the KCRV derived from the other six results was 0.9161 with associated standard uncertainty of 0.0027.

**Fig. 15:**
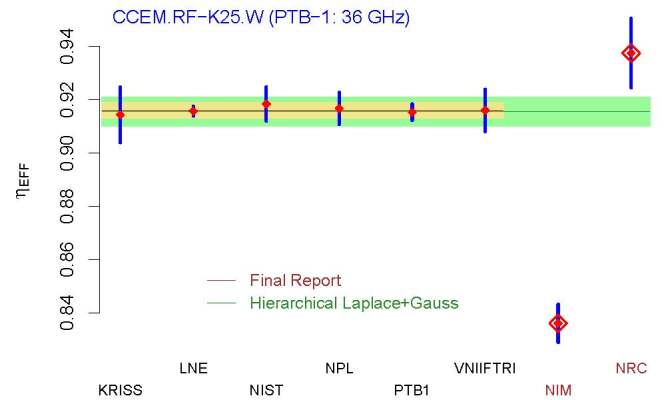
Measurement results for the effective efficiency of traveling standard PTB-1 from CCEM.RF-K25.W, at frequency 36 GHz. The final report excludes the results from NIM and NRC because they are "statistical outliers." The Decision Tree recommends the *Hierarchical Laplace* + *Gauss* procedure for the full data set, which accommodates the results from NIM and NRC while protecting the KCRV from their influence. The larger uncertainty associated with the alternative KCRV (represented by half the height of the pale green band) is attributable more to the model recognizing that the estimate of dark uncertainty is based on a small number of measurement results, than to the accommodation of the results from NIM and NRC. The acronyms and initialisms designating the participants are defined in Ref. [65].

**Fig. 16:**
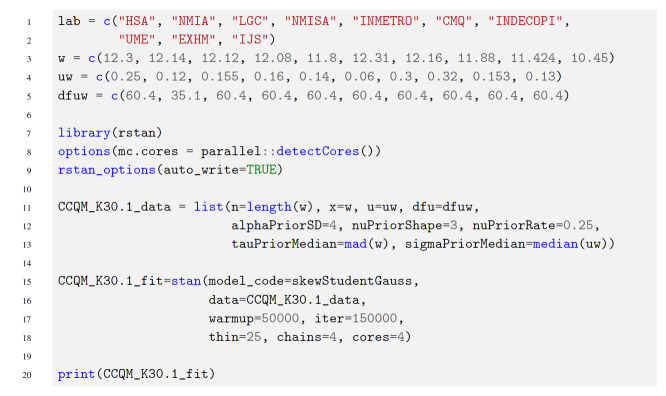
R code that applies the *Hierarchical Skew Student* + *Gauss* model as implemented in Fig. 17 to the example discussed in Sec. 4.5, using facilities available in R package rstan [55]. This R code assumes that the Stan code from Fig. 17 has been assigned to variable skewStudentGauss as a character string. The execution of this code on a Dell Optiplex 7020 computer with an Intel Core i7-4790 CPU with four cores running at 3.60 GHz, takes about 15 min.

**Fig. 17:**
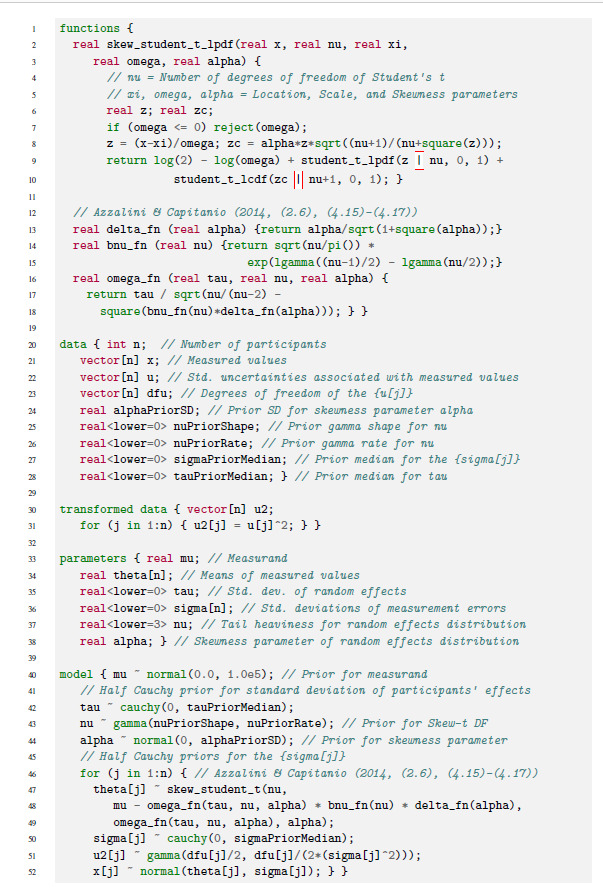
Stan code implementing the *Hierarchical Skew Student* + *Gauss* model.

The Decision Tree examined all eight results and concluded that they were mutually inconsistent, and that the measured values appear to be a sample from a symmetric but non-Gaussian distribution: therefore, it recommended the *Hierarchical Laplace* + *Gauss* procedure. The resulting KCRV was 0.9156 with associated standard uncertainty of 0.0052. Since this estimate of *η*_EFF_ and the corresponding estimate from the final report are statistically indistinguishable, we conclude that the Decision Tree manages to reproduce the result in the KC's final report while honoring principle (P1) of Sec. 2 (Fig. 15).

## 5 Conclusions and Recommendations

The recommendations provided by the Decision Tree are driven by reasonable principles of metrological practice and rest upon rigorous, proven methods of applied statistics that are widely used in fields where interlaboratory studies and meta-analyses are undertaken routinely.

However, the statistical tests recommended for use at each node of the Decision Tree may not produce reliable results when the number of participants in the KC is very small (say, less than 5). In such cases, professional judgment or lessons learned from similar but larger data sets may be needed to navigate the Decision Tree confidently.

An integrated implementation of the whole Decision Tree as a Shiny App (https://shiny.rstudio.com/) is under development. A future version of the Decision Tree may capitalize on the accumulated experience with KCs in a particular area (say, inorganic analysis), to develop meaningful prior information about the relative heterogeneity that is likely to be encountered, and then to take this historical information into account as an aid in deciding whether there is statistically significant heterogeneity.

Reliance on the Decision Tree avoids the need, often felt by CCs and by other bodies that organize interlaboratory studies, to set aside measurement results that do not seem to ft with the bulk of the others, but that cannot easily be dismissed on substantive grounds. The example in Sec. 4.7, on the effective efficiency of a power sensor, illustrates this cogently.

The Decision Tree also is able to cope with situations where the "standard" assumptions are not met (in particular, the assumptions of symmetry or Gaussian shape for the laboratory effects), and for which sub-optimal choices have been made: for example, computing the KCRV as the median of the measured values, ignoring the reported uncertainties and glossing over any dark uncertainty that may be present.

The methods implemented in the leaves of the Decision Tree all properly account for correlations between the KCRV and individual measurement results, which are much too often neglected in Final Reports from KCs. This is facilitated by systematically using either the statistical bootstrap, or the results of Markov Chain Monte Carlo sampling [66] for uncertainty evaluations. These general purpose Monte Carlo methods are versatile statistical tools that address the challenges posed by such correlations, and that also effectively propagate evaluations of dark uncertainty, especially as they impact the DoEs.

The expanded uncertainties in the DoEs presented in the foregoing often were considerably larger than their counterparts in the Final Reports. In some cases this was attributable to the inclusion of "discrepant" measurement results in the calculation of the KCRV, but the most persistent reason for the differences between DoEs in Final Reports and in the present, alternative treatments, is whether dark uncertainty was propagated to the DoEs, and how.

In addition to evaluating and propagating dark uncertainty properly, the methods implemented in the leaves of the Decision Tree recognize and take into account the fact that the evaluation of the contributions of dark uncertainty is often based on a very small effective number of degrees of freedom (which equals the number of participants in the KC, minus 1).

The effects that proper consideration of dark uncertainty has upon the DoEs are particularly clear in the examples of Sec. 4.4, Sec. 4.5, and Sec. 4.6.1, generally inducing a more guarded assessment of the significance of apparent differences between measured values and the KCRV.

The examples in Sec. 4.6 concerning measurements of properties of radionuclides, show how the Decision Tree, while a general-purpose aid, can successfully address cases for which specialized solutions had been developed.

The Decision Tree is a potentially useful guide for modeling and reducing the measurement results obtained in a KC, and it is likely to reduce the time and effort that typically have been expended in such tasks. We believe that it offers an approach that is much more defensible metrologically than several alternatives that have been employed historically, as reviewed in the examples in Sec. 4.

However, the Decision Tree provides no guidance as to which measurement results to include and which to set aside when characterizing the KCRV: this is a substantive task and challenge that only the participants can and should address, relying on their expert knowledge.

## 6 **Appendix: Computer Codes for Hierarchical Skew Student** + **Gauss Model**

This appendix provides detailed information about the *Hierarchical Skew Student* + *Gauss* model introduced in Sec. 3.2.5, and lists R (Fig. 16) and Stan (Fig. 17) computer codes that implement this model, which may be used to reproduce the results from Sec. 4.5.

The parameters of primary interest are the overall mean, *µ* in Eq. (1), the standard deviation that quantifies dark uncertainty, *τ*, and possibly the true values, {*σ*_*j*_}, of the reported uncertainties, when these are based on specified, finite numbers of degrees of freedom. These {*σ*_*j*_} are the standard deviations of the participant-specific measurement errors, the {*ε*_*j*_} in Eq. (1).

The model also includes two parameters of secondary interest: the skewness parameter, *α*, of the probability distribution of the participants' effects, which are the *{ℷ_j_}* in Eq. (1), and the number of degrees of freedom, *v*, of the underlying Skew Student distribution. The parameter *α* controls the asymmetry of this distribution, and *v* controls the heaviness of its tails.

The interplay between *v* and *α* is particularly delicate because a large value for *v* combined with an *α* with small absolute value can produce results similar to those obtained using a small *v* and an *α* with large absolute value. Scientific judgment will be the best guide for how to balance the heaviness of the tails (controlled by *v*) and the degree of asymmetry (controlled by *α*) that may reasonably be expected for the distribution of the participants' effects.

Since the *Hierarchical Skew Student* + *Gauss* model is a Bayesian model, it also comprises probability distributions for all the parameters that figure in the model. These prior distributions include parameters of their own, called *hyper-parameters*, and the values of several of these need to be specified in each instance of application.

Five hyper-parameters require careful tuning, specific to each dataset the model will be fitted to: (1) prior median of *τ*; (2) prior median for the {*σ*_*j*_}; (3) shape and (4) rate of the gamma prior distribution for *v*; and (5) prior standard deviation of *α*. The choices made in the R code of Fig. 16 are appropriate for the measurement results discussed in Sec. 4.5, but they may not be the best choices for other sets of measurement results.

We recommend that, for general use, alphaPriorSD (in Line 12 of Fig.16) should be set equal to 4. The values assigned to nuPriorShape and to nuPriorRate in Line 12 of Fig.16, 3 and 0.25 respectively, may also prove to be adequate for general use: they suggest 12 as number of degrees of freedom expected *a priori* and hence moderately heavy tails for the skew-*t* distribution of the participants' effects.

Figure 17 lists a stand-alone implementation of the *Hierarchical Skew Student* + *Gauss* model formulated in the Stan [53] language. Figure 16 illustrates its application to the measurement results from CCQM-K30.1 in Table 5. The following explanations pertain to the numbered lines of Stan code in Fig. 17:

**Lines 33 and 40** The true value of the measurand, *µ*, is *ξ* + *ωδ*b_v_*, where ξ* is the location parameter of the Skew Student distribution, *ω* is its scale parameter, $\delta =\alpha \sqrt{1+\alpha^{2}}$[51, Eq. (2.6)], and $b_{v}=\sqrt{v}\Gamma ((v-1)/2)/(\sqrt{\pi }\Gamma (v/2))$ [51, Eq. (4.15)]. The prior distribution selected for *µ* is a proper but non-informative Gaussian distribution, centered at 0, with very large standard deviation.

**Lines 35 and 42** *$\tau =\omega \sqrt{v/(v-2)-{\left(\delta b_{v}\right)}^{2}}$* is the standard deviation of the participants' effects, which are the {ℷ_*j*_} in Eq. (1). Its prior distribution is half-Cauchy [54] with median tauPriorMedian, which the R code of Fig. 16 sets equal to the median absolute deviation from the median of the measured values.

**Lines 36 and 50** The reported standard uncertainties are regarded as estimates of standard deviations {*σ_j_*}, based on finite, but possibly large numbers of degrees of freedom, which are the entries of dfuw in Line 5 of the R code in Fig. 16. These numbers of degrees of freedom correspond to the coverage factors *k* listed in Table 5.

**Lines 37 and 43** The number of degrees of freedom, *v*, has a gamma prior distribution truncated at 3, whose shape and rate parameters have values specified in Line 12 of the R code in Fig. 16. The truncation at 3 ensures that the distribution of the participants' effects, {*λ_j_*}, has a finite, but otherwise unrestricted skewness coefficient [51, p. 104].

**Lines 38 and 44** The skewness parameter *α* has a Gaussian prior distribution centered at 0 with standard deviation specified in Line 12 of the R code in Fig. 16.

**Lines 47-49** The location parameter, *ξ*, of the Skew Student distribution does not appear explicitly in the Stan code, but its value is computed as part of the specification of the probability distribution of the true value, *θ_j_* = *µ* + *λ_j_*, measured by participant *j*, where *λ_j_* is the participant's effect, for *j* = 1, *. . ., n*.

**Line 51** Considering that each *u*^2^(*x_j_*) is part of the data, and that it is based on a finite number of degrees of freedom, it is modeled as an outcome of a rescaled chi-squared random variable, consistently with the assumption that the participant-specific measurement errors are Gaussian.

**Line 52** The measured values are modeled as outcomes of Gaussian random variables, conditionally upon the values of the {*θ_j_*} and of the {*σ_j_*}, which are estimated in the process. The estimates of the {*σ_j_*} take into account the fact that the reported uncertainties are based on finite (even if possibly very large) numbers of degrees of freedom.
